# The impact of donor liver fibrosis on the outcomes of patients who undergo liver transplant: a cohort study from UNOS database

**DOI:** 10.3389/fmed.2025.1547200

**Published:** 2025-04-10

**Authors:** Congwen Bian, Jiajiao Luo, Ruizhu Tang, Hanfei Huang, Zhong Zeng

**Affiliations:** ^1^Department of Organ Transplantation, The First Affiliated Hospital of Kunming Medical University, Kunming, China; ^2^Department of Surgery, Li Ka Shing Faculty of Medicine, The University of Hong Kong, Hong Kong, Hong Kong SAR, China; ^3^Department of Clinical Laboratory, The First Affiliated Hospital of Kunming Medical University, Kunming, China

**Keywords:** donor fibrosis, graft failure, transplant outcome, rejection, liver transplantation

## Abstract

**Background:**

The increasing prevalence of fibrosis in donor livers raises concerns about its impact on post-transplantation outcomes, though this relationship remains unclear. This study aims to assess the effect of donor liver fibrosis on patient and graft survival following liver transplantation.

**Methods:**

Data from the UNOS-SRTR registry (1987–2024) were analyzed, focusing on patients who received liver transplants with biopsy-proven fibrosis. The cohort was stratified based on fibrosis grade, and outcomes were compared using Cox regression and Kaplan–Meier survival analysis. Competing risk models were applied to assess specific causes for graft failure, and subgroup analyses explored the sensitivity of fibrosis on transplant outcomes.

**Results:**

Of the 22,897 patients, 17,926 received non-fibrotic grafts, and 4,971 had grafts with varying fibrosis grades. Donor fibrosis was associated with donor age, steatosis, and portal infiltrate, generally affecting those in better overall condition. Significant differences were observed in patient survival (*p* = 0.001) and graft survival (*p* = 0.002) between the fibrosis and non-fibrosis groups. Further analysis revealed that fibrosis increased the risk of malignancy (*p* = 0.028), cardiovascular disease (*p* = 0.017), and respiratory failure (*p* = 0.033), but showed lower rejection rates at six months and one year. Sensitivity analyses confirmed fibrosis as an independent risk factor, with varying effects in subgroups.

**Conclusion:**

Donor liver fibrosis significantly impacts post-transplant outcomes, notably increasing the risk of all-cause mortality and graft failure. Specific causes of death, such as malignancy and cardiovascular disease, were more prevalent in recipients of fibrotic grafts, highlighting the need for further research to refine donor selection criteria.

## Introduction

Liver transplantation (LT) remains the most effective treatment for end-stage liver disease, offering patients a chance for long-term survival and improved quality of life (1), However, the success of this procedure mainly depends on the quality of the donor liver (2). In recent years, the disparity between organ availability and demand has contributed significantly to waitlist mortality and has thereby led to increased utilization of marginal or extended criteria donor (ECD), with the increasing prevalence of liver fibrosis in donor organs, which now has been emerged as a significant challenge for transplantation (3, 4). Fibrosis, which results from chronic liver injury, leads to the accumulation of fibrotic tissue in the liver and has become more common due to various reasons, the elder donor population (5)and rising incidences of metabolic disorders such as obesity, diabetes, and metabolic dysfunction associated with steatohepatitis (MASH) (6–8), While liver, including patient survival and graft function, remains insufficiently studied.

Previous studies have focused on various donor characteristics, such as age, body mass index (BMI) (9, 10), and the presence of steatosis (11, 12), but have largely overlooked the role of fibrosis itself. Fibrosis progression after transplant is often considered a negative prognostic factor (13, 14), which leads to early graft loss and patient death. However, the impact of donor fibrosis is controversial, the lack of clear and solid evidence creates confusion when determining whether to use fibrotic livers and selecting appropriate patients. As the demand for donor organs continues to exceed supply, understanding the risks and outcomes associated with using fibrotic grafts is crucial for optimizing transplant success and ensuring better long-term outcomes for recipients (Table 1).

**Table 1:** Baseline characteristic of donor and recipients between fibrosis group and the primary outcomes.

Characteristics	All	*N*	Fibrosis group		*P*_value
			No (*n* = 17,926)	Yes (*n* = 4,971)	
Donors					
Age_donor (Mean, SD, yrs)	47.6 (14.7)	22,897	47.3 (14.9)	48.6 (13.9)	<0.001
Gender_donor (Male, %)	12,946 (56.5%)	22,897	9,968 (55.6%)	2,978 (59.9%)	<0.001
BMI_donor	30.1 (7.56)	22,861	30.1 (7.58)	30.1 (7.50)	0.534
HCV (*n*, %)	2,170 (9.49%)	22,878	1,683 (9.40%)	487 (9.80%)	0.406
CMV (*n*, %)	14,950 (65.5%)	22,820	11,703 (65.5%)	3,247 (65.5%)	1
HBV (*n*, %)			1,343 (7.50%)	539 (10.8%)	<0.001
Portal inflammation		22,897			<0.001
Mild	10,596 (46.3%)		7,688 (42.9%)	2,908 (58.5%)	
Moderate_servere	1,637 (7.15%)		844 (4.71%)	793 (16.0%)	
Macro_steatosis (mean, SD, %)	8.71 (11.9)	22,897	8.20 (11.5)	10.5 (12.9)	<0.001
Micro_steatosis (mean, SD, %)	9.75 (15.7)	22,897	9.36 (15.7)	11.1 (15.7)	<0.001
Cold_ischemia time (mean, SD, hrs)	6.24 (2.36)	22,822	6.26 (2.38)	6.18 (2.30)	0.031
Recipients					
Age (Mean, SD, yrs)			56.2 (10.7)	57.0 (10.3)	<0.001
BMI (Mean, SD, Kg/m^2^)			29.2 (5.94)	29.2 (5.82)	0.828
Ethnicity (*n*, %)		22,897			<0.001
Non-Hispanic/Non-Latino	19,408 (84.8%)		15,094 (84.2%)	4,314 (86.8%)	
Hispanic/Latino	3,489 (15.2%)		2,832 (15.8%)	657 (13.2%)	
Gender (Male, %)	15,378 (67.2%)	22,897	11,980 (66.8%)	3,398 (68.4%)	0.044
ABO incompatible match (n, %)	362 (1.58%)	22,897	288 (1.61%)	74 (1.49%)	0.407
TIPS (n, %)	2,425 (10.8%)	22,443	1913 (10.9%)	512 (10.5%)	0.485
Portal vein thrombosis (n, %)	3,230 (14.1%)	22,837	2,517 (14.1%)	713 (14.4%)	0.586
Creatinine (mean, SD, mg/dl)	1.52 (1.37)	22,897	1.53 (1.36)	1.49 (1.40)	0.113
Bilirubin (mean, SD, mg/dl)	7.40 (9.71)	22,897	7.68 (9.94)	6.37 (8.78)	<0.001
INR (mean, SD)	1.90 (1.14)	22,897	1.93 (1.19)	1.80 (0.91)	<0.001
Albumin (mean, SD,g/dl)	3.19 (0.69)	22,897	3.19 (0.69)	3.18 (0.68)	0.463
Dialysis (*n*, %)	3,030 (13.3%)	22,773	2,475 (13.9%)	555 (11.2%)	<0.001
Encephalopathy (*n*, %)		22,897			0.701
I-II	20,208 (88.3%)		15,829 (88.3%)	4,379 (88.1%)	
III-IV	2,689 (11.7%)		2097 (11.7%)	592 (11.9%)	
Ascites (*n*, %)		22,897			0.11
I-II	15,309 (66.9%)		11,938 (66.6%)	3,371 (67.8%)	
III-IV	7,588 (33.1%)		5,988 (33.4%)	1,600 (32.2%)	
MELD_score (mean, SD)	6,945 (30.3%)	22,897	22.9 (10.3)	21.4 (9.59)	<0.001
Diabetes (*n*, %)	6,945 (30.3%)	22,897	5,359 (29.9%)	1,586 (31.9%)	0.007
Life support (*n*, %)	1,530 (6.68%)	22,897	1,245 (6.95%)	285 (5.73%)	0.003
HBV (*n*, %)	743 (3.24%)	22,897	604 (3.37%)	139 (2.80%)	0.08
HCV (*n*, %)	5,112 (22.7%)	22,560	3,901 (22.1%)	1,211 (24.7%)	<0.001
HCC (*n*, %)	6,591 (28.9%)	22,823	5,111 (28.6%)	1,480 (29.8%)	0.097
Outcomes					
Hospital_stay after LT (mean, SD,days)	3,230 (14.1%)	22,837	15.7 (21.7)	15.7 (24.6)	0.974
Overall graft survival (*n*, %)	4,532 (19.8%)	22,897	3,507 (19.6%)	1,025 (20.6%)	0.102
Graft survival time (mean, SD, days)	1,285 (857)	22,897	1,303 (864)	1,220 (828)	<0.001
Patient survival (*n*, %)	4,101 (17.9%)	22,897	3,169 (17.7%)	932 (18.7%)	0.085
Patient survival time (mean, SD,days)	1,287 (857)	22,897	1,305 (865)	1,222 (828)	<0.001
Graft failure from malignancy (*n*, %)	665 (2.90%)	22,897	506 (2.82%)	159 (3.20%)	0.178
Graft failure for CVD (*n*, %)	582 (2.54%)	22,897	439 (2.45%)	143 (2.88%)	0.100
Graft failure for infection (*n*, %)	599 (2.62%)	22,897	471 (2.63%)	128 (2.57%)	0.877
Graft failure for respiratory failure (*n*, %)	249 (1.09%)	22,897	182 (1.02%)	67 (1.35%)	0.054
Early allograft failure (EAF) (*n*, %)	1,180 (5.15%)	22,897	914 (5.10%)	266 (5.35%)	0.499
Re_transplantation (*n*, %)	565 (2.47%)	22,897	445 (2.48%)	120 (2.41%)	0.823
Rejection in 6 months (*n*, %)	1,579 (8.61%)	18,346	1,292 (9.02%)	287 (7.13%)	<0.001
Rejection in 1 year (*n*, %)	1854 (10.4%)	17,784	1,500 (10.8%)	354 (9.03%)	0.001
Liver related Graft Failure (*n*, %)	1,063 (4.64%)	22,897	837 (4.67%)	226 (4.55%)	0.747
Followup_time (mean,SD,days)	1,285 (857)	22,897	1,303 (864)	1,220 (828)	<0.001

Data are presented as No. (%) or mean SD. HCC, hepatocellular carcinoma; HBV, hepatitis B virus; HCV, hepatitis C virus; CMV, cytomegalovirus; BMI, body mass index; MELD, model for endstage liver disease.

The objective of this study is to evaluate the impact of donor liver fibrosis on post-transplant outcomes, specifically focusing on all-cause mortality and graft survival. Using data from the UNOS registry (15), this study aims to explore whether the presence and severity of fibrosis in donor livers are independent risk factors for adverse transplant outcomes for a long-term follow-up. Additionally, this study investigates the relationship between fibrosis and specific causes of mortality, such as malignancy and cardiovascular disease, while also assessing potential protective factors, such as lower rejection rates within the first year after transplantation.

**Figure 1:**
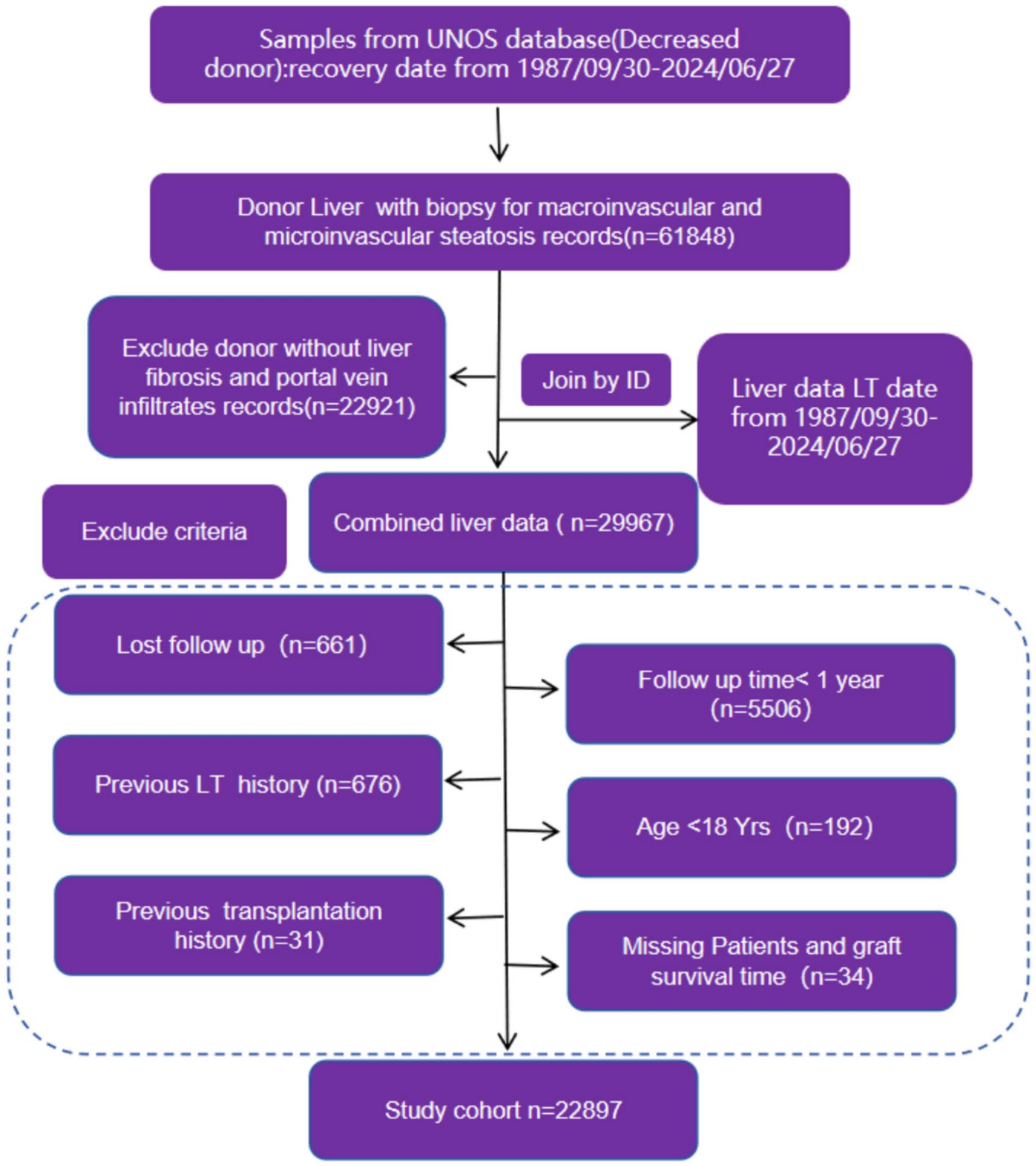
The flow chat of sample include and exclude criteria.

To achieve these aims, this study analyzed a large cohort of liver transplant recipients using advanced statistical methods. Patients were stratified based on the histologic grade of fibrosis in the donor grafts, and outcomes were compared using Cox proportional hazards models and Kaplan–Meier survival analysis. Further analyses included competing risk models to assess specific causes of mortality, along with subgroup sensitivity analyses to evaluate the robustness of fibrosis as an independent risk factor. By leveraging the extensive data available from the UNOS-SRTR registry, this study provides a comprehensive assessment of the risks associated with receiving fibrotic donor grafts and contributes to refining donor selection criteria (Figure 1).

**Figure 2:**
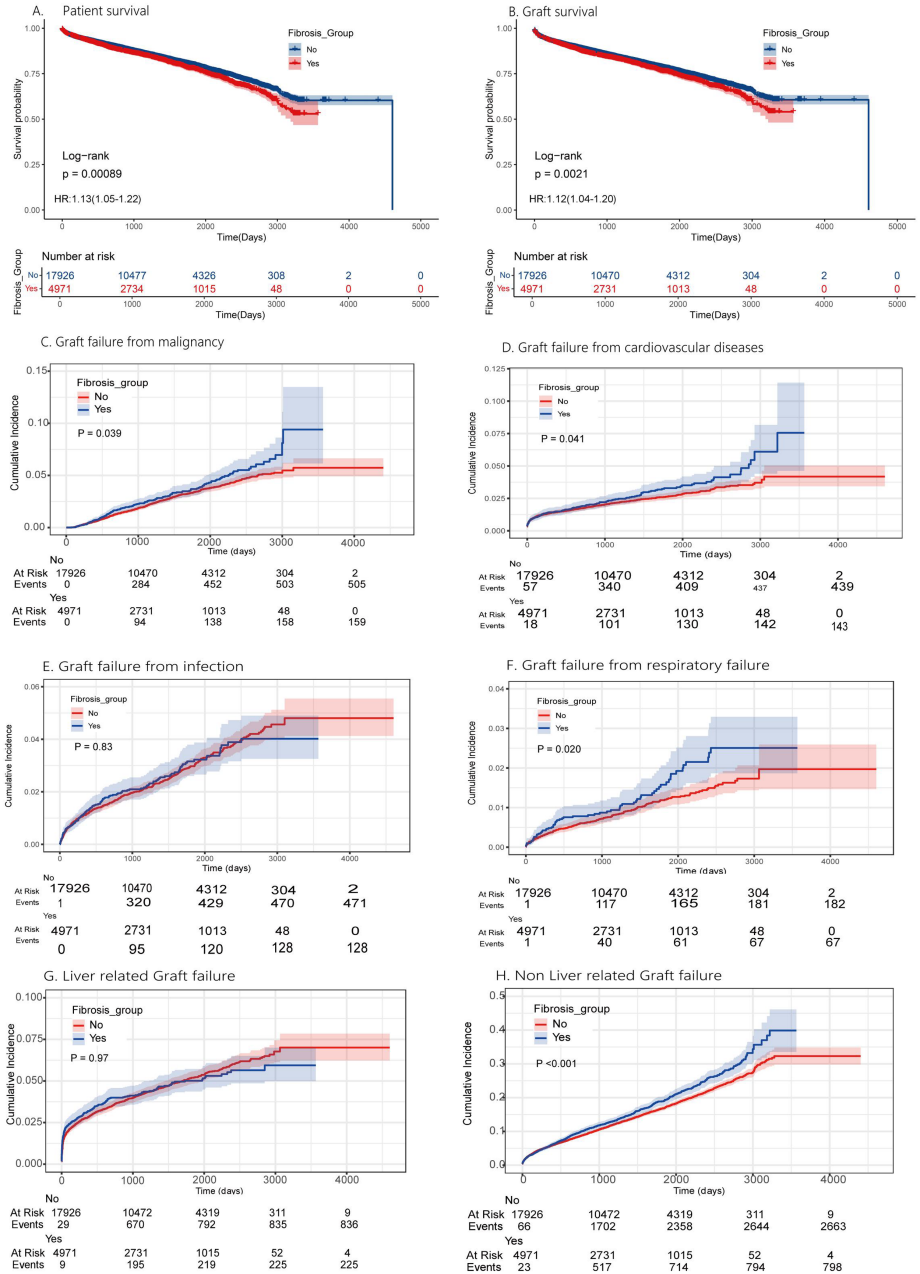
Kaplan–Meier survival and competing risk model for liver transplantation outcomes in fibrosis group. The patients and grafts overall survival are presented in panel A and B, with *P*-value <0.001 and *P*-value <0.01.The impact of fibrosis for special cause of graft failure by competing risk model presented in panel C-H, with significant *P*-value in cardiovascular disease, malignancy, respiratory failure.

To be short, this study seeks to clarify the role of donor liver fibrosis in influencing post-transplant outcomes. By identifying the specific risks and potential benefits associated with using fibrotic grafts, this research has the potential to instruct clinical decision-making and improve the allocation of donor organs. Ultimately, the findings will help guide transplant professionals in balancing the risks of fibrosis with the pressing need for donor organs, thereby optimizing outcomes for liver transplant recipients (Figure 2).

## Methods

### Patients selection

**Figure 3:**
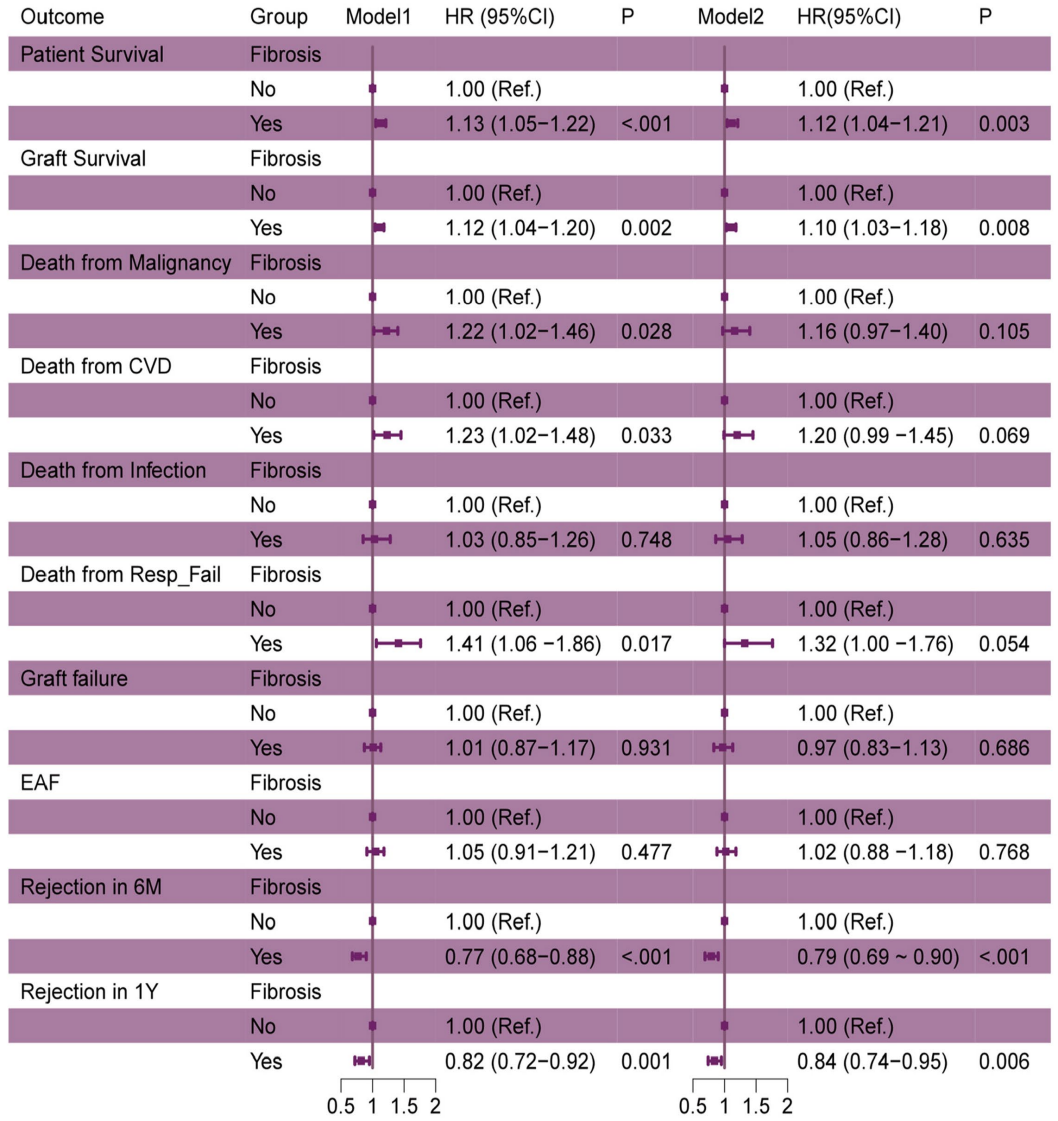
The association between fibrosis and LT outcomes. OR, odds ratio, CI, confidence interval. Model1: Crude. Model2: Adjust: macro_steatosis, micro_steatosis, cold ischemia time, age, gender, bilirubin, dialysis, encephalopathy, ascites, MELD_score, diabetes, life support, HCC.

This study utilized data from the United Network for Organ Sharing (UNOS) Scientific Registry of Transplant Recipients (SRTR), which includes liver transplant recipients between September 30, 1987, and April 1, 2024. The primary focus was on deceased donor liver transplant cases. The initial dataset comprised 291,377 transplant cases, from which the study cohort was refined based on specific exclusion criteria. Patients were excluded if they had incomplete liver biopsy data for macrovascular and microvascular steatosis, missing liver fibrosis or portal vein infiltrates records, missing key variables such as Patient or Graft survival time, or if they were under the age of 18 or had undergone prior liver transplants or other organ transplantation. In order to assess the outcome more precisely we also exclude a follow-up time of less than one year. After applying these criteria, the final study cohort included 22,898 patients. This cohort was further stratified based on the histologic grade of fibrosis, with the aim of comparing post-transplant outcomes (Figure 3).

### Outcomes

**Figure 4:**
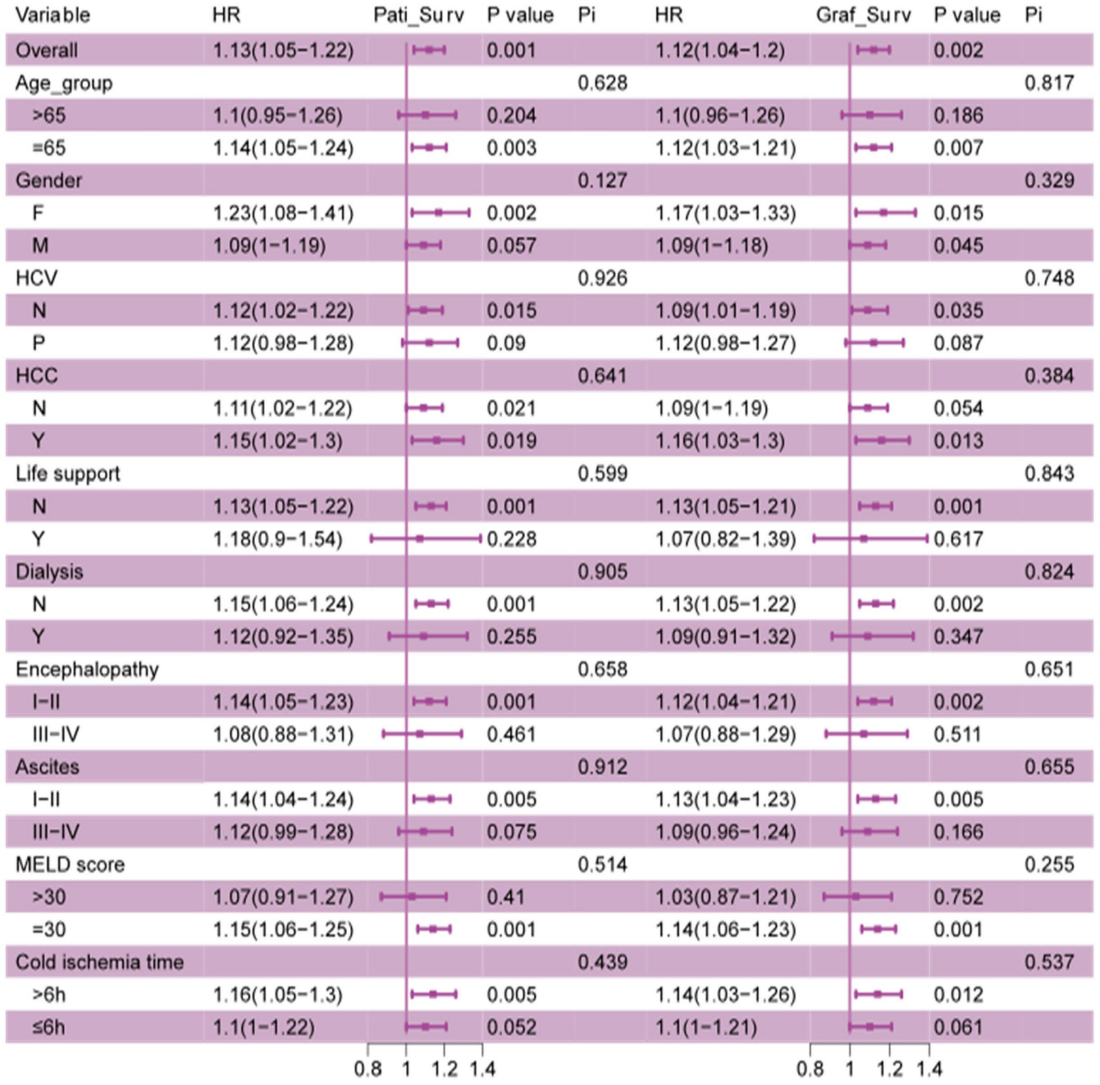
The subgroup analysis of impact of fibrosis on recipients and grafts survival. Stratified associations between fibrosis and patients and grafts survival. All ORs were calculated by adjusting for Cold ischemia time, age, gender, HCC, dialysis, encephalopathy, ascites, MELD_score, diabetes, life support.

The primary outcomes of interest were all-cause mortality and graft survival. Secondary outcomes included specific causes of mortality, such as malignancy, cardiovascular disease, and respiratory failure, as well as early rejection rates within six months and one-year post-transplant. Historically variable factors, such as donor age, the presence of steatosis, and portal infiltrates, were also analyzed to assess their association with fibrosis and transplant outcomes. These factors provided context to better understand how donor conditions influenced the recipient’s survival and graft function after the transplant (Figure 4).

### Statistical analysis

Statistical analyses were conducted using Cox proportional hazards regression models to evaluate the impact of donor liver fibrosis on patient survival and graft survival. Kaplan–Meier (KM) survival and competing risk models were used to assess specific causes of mortality, such as malignancy and cardiovascular disease while accounting for the competing risk of death from other causes. Subgroup analyses were performed to explore the sensitivity of fibrosis as an independent risk factor across various recipient demographics and clinical conditions. All statistical analyses were conducted using statistical software, and a *p*-value of less than 0.05 was considered statistically significant.

## Results

### Baseline of donor and recipient characteristic

The study analyzed 22,897 liver transplant recipients, comparing those who received non-fibrotic grafts (*n* = 17,926) with those who received fibrotic grafts (*n* = 4,971). Donors’ average age was higher in the fibrosis group (48.6 vs. 47.3 years, *p* < 0.001), and gender differences were notable, with 59.9% male in the fibrosis group versus 55.6% in the non-fibrotic group (*p* < 0.001). Macro-steatosis in the liver also differed significantly, averaging 10.5% fatty change in the fibrosis group versus 8.2% in the non-fibrotic group with p < 0.001, the same trend observed in micro-steatosis with *p* < 0.001. There were no differences in HCV and HBV infection between the two groups. About the recipient’s characteristics, older age (57.0 vs. 56.2 years, *p* < 0.001) and higher MELD scores (22.9 vs. 21.4, *p* < 0.001) were seen in the fibrosis group. The liver function at transplant and other indicators like total bilirubin (6.37 vs. 7.68, *p* < 0.001) and INR (1.80 vs. 1.93, p < 0.001) showed significant disparities between the two groups. In terms of the outcome, notably, rejection rates within six months were lower in the survival group (7.13% vs. 9.02%, *p* < 0.001), and one-year rejection rates were also lower (9.03% vs. 10.8%, *p* = 0.001). The no-fibrosis group demonstrated longer patient survival times (1,305 vs. 1,222 days, *p* < 0.001) and graft survival times (1,303 vs. 1,220 days, *p* < 0.001). However, hospital stay length and rates of failure due to malignancy, cardiovascular disease, infection, and respiratory failure showed no significant differences. This data shows us the main features of donor and recipient characteristics in two groups. Providing insights for the reason of fibrosis and donor allocation in matching suitable recipients.

The KM survival analysis showed that recipients of fibrotic grafts had significantly worse patient survival (HR 1.42, 95%CI:1.05–1.22, *p* = 0.039) and graft survival (HR 1.36, 95%CI:1.04–1.22 *p* = 0.041). By competing risk model analysis we found more precise results. Fibrosis was linked to higher mortality from cancer (*p* = 0.039), cardiovascular disease (*p* = 0.041), and respiratory failure (*p* = 0.020), these findings are consistent with previous research suggesting that fibrosis can impair organ function, potentially contributing to these specific causes of death. Infection and liver-related graft failure rates were similar between groups, but non-liver-related graft failure-related deaths were significantly higher in the fibrosis group (*p* < 0.001). These results indicate that donor liver fibrosis significantly impacts post-transplant outcomes.

The multivariate regression analysis revealed that donor liver fibrosis significantly impacts transplant outcomes. In a crude model (Model 1), recipients of fibrotic grafts had a 12–13% higher risk of death (HR 1.13, 95%CI: 1.05–1.22,*p* < 0.001), which remained significant after adjusting for 10 covariates in Model 2 (HR 1.12, 95%CI:1.04–1.21, *p* = 0.003). The risk of all causes of graft failure showed similar increases (Model 1: HR 1.12, 95%CI: 1.04–1.20, *p* = 0.002; Model 2: HR1.10, 95%CI: 1.03–1.18,*p* = 0.008). For special causes of graft failure, donor hepatic fibrosis was also associated with a higher risk of death from cardiovascular disease and respiratory failure. However, the latter was borderline significant in the adjusted model (*p* = 0.054). Interestingly, fibrosis was linked to lower rejection rates at 6 months (HR 0.77, 95%CI: 0.68–0.88, *p* < 0.001) and 1 year (HR 0.82, 95%CI: 0.72–0.92, *p* = 0.001) but with no different in early allograft failure. The 10 covariates added for adjustment included donor and recipient characteristics such as age, gender, cold ischemia time, and MELD score, which showed that fibrosis affects outcomes beyond these factors, particularly influencing survival and rejection rates.

The subgroup analysis explored the sensitivity of donor liver fibrosis on transplant outcomes across different recipient groups. Overall, fibrosis was associated with worse overall patients and graft survival, but its impact varied across subgroups. Recipients aged less than 65 experienced a significant increase in mortality risk, with HR 1.14 (95%CI: 1.05–1.24, *p* = 0.003) for patient survival and HR 1.12 (95%CI: 1.03–1.21, *p* = 0.007), while those over 65 did not been affected by fibrosis. Both genders showed higher risks, with males being more vulnerable for fibrosis, for patient and graft survival, with *p* = 0.002 and p 0.015, respectively. HCV-positive recipients were more sensitive to fibrosis (HR 1.15, 95%CI: *p* = 0.019) compared to HCV-negative (HR 1.12, 95%CI: *p* = 0.021). Additionally, patients with hepatocellular carcinoma (HCC) had increased risk (HR 1.15, 95%CI: *p* = 0.005), and those with MELD scores >30 were particularly affected (HR 1.16, 95%CI: *p* = 0.001). Also, we found that those who have a better status of pre-transplantation may be affected mostly by fibrosis, without life support, dialysis, grade I-II ascites, and grade I-II encephalopathy. There is no interaction among fibrosis and all subgroups with no significant *p*-value.

## Discussion

This study demonstrates that donor liver fibrosis significantly impacts post-transplant prognosis, both short-term and long-term outcomes, particularly patient and graft survival. To our knowledge, this is the largest sample size of patients analyzing hepatic fibrosis about LT outcomes. Additionally, this study is novel in the application of a competing risk survival model and subgroup analysis to further analyze various causes of Mortality. This allows for the identification of particularly vulnerable recipients for fibrotic grafts and improves the precise management of post-LT.

Our data revealed that recipients of grafts with hepatic fibrosis had significantly lower survival rates compared to those without fibrosis. Despite histological examination helping assess organ viability, the true impact of fibrosis on recipient outcomes remains unclear. In contrast to Wadhera et al. (16) findings, our study shows that recipients of fibrotic livers had a 12–13% higher risk of death and graft failure, even after adjusting for important covariates. Similar findings were reported by D’Errico (18), suggesting that fibrosis independently diminishes transplant long-term survival and underscores the importance of careful donor selection and vigilant monitoring of recipients receiving fibrotic grafts (17, 18). Regarding the specific cause of graft failure and short-term outcomes, including early allograft failure (EAF), and allograft rejection (AR). We see no difference in EAF incidence, but a solid difference in allograft rejection in half and one year, with a lower incidence in the fibrosis group. Although previous studies have not widely recognized the association between rejection and pre-existing fibrosis, emerging evidence suggests that antibody mediated rejection may drive graft fibrosis progression post-transplant (19–22). On the other hand, whether there is the possibility of local immune environment formation due to higher portal inflammation making a major contribution is unknown, further exploration needs to be conducted for validation.

The subgroup analysis offers valuable insights into how recipient characteristics alter the effect of donor liver fibrosis on outcomes. Younger recipients (≤65 years) showed a 14% higher mortality risk, while older patients were not similarly affected. This indicates that younger patients might be more vulnerable to the adverse effects of fibrosis, potentially due to higher expectations for graft longevity and function. Thus, age should be a key consideration in determining the suitability of fibrotic grafts (23). Additionally, gender differences were observed, with male recipients experiencing slightly higher risks than females, potentially reflecting physiological differences or underlying comorbidities (24). HCV-positive recipients were more sensitive to the effects of fibrosis, experiencing greater risks than their HCV-negative counterparts. This aligns with the understanding that hepatitis C exacerbates liver graft complications, reinforcing the need for specialized post-transplant care in HCV-positive patients, which is consistent with the recurrence of HCV and will promote the progression of liver allograft fibrosis (25, 26). Furthermore, patients with high MELD scores (>30) and those diagnosed with hepatocellular carcinoma (HCC) experienced worse outcomes with fibrotic grafts, indicating that fibrosis amplifies their pre-existing vulnerabilities. These findings highlight the importance of personalized donor-recipient matching, particularly for high-risk groups.

This study demonstrates that donor liver fibrosis significantly impacts post-transplant outcomes, increasing the risks of patient mortality and graft failure. Fibrosis independently influences survival, especially in younger, HCV-positive, and high-MELD recipients, who are more vulnerable to its effects. However, the use of fibrosis reduced the number of deaths on the waiting list, besides the progression is not definitely to be worse. It reported that 30% of liver fibrosis alleviated, 40% stayed stable and 30% elevated (16), these indicated the complexity of liver fibrosis and good post-transplantation make a difference (27). Additionally, fibrosis was associated with lower rejection rates at six months and one year post-transplant. These findings emphasize the importance of personalized donor-recipient matching and targeted post-transplant care for high-risk patients.

However, limitations should be acknowledged. First, the study’s retrospective design and reliance on registry data introduce the possibility of bias, particularly regarding incomplete or missing records. Variability in histologic grading across transplant centers could also impact data consistency, as fibrosis staging may differ by pathologist interpretation. Additionally, although lower rejection rates were observed in recipients of fibrotic grafts, the mechanisms behind this finding are not fully understood. One hypothesis is an altered immune response triggered by the fibrotic liver environment, which may merit further exploration. Despite these limitations, this study provides valuable clinical insights, underscoring the need for personalized donor-recipient matching and vigilant post-transplant management in high-risk patients.

## Conclusion

In conclusion, this study demonstrates that donor liver fibrosis significantly compromises transplant outcomes, particularly among younger recipients and HCV-positive patients. Furthermore, decreased rejection rates in recipients with fibrotic grafts give additional information about putative immunological processes. These findings highlight the importance of thorough donor screening and individualized matching to improve transplant outcomes. Future research should aim to explore the underlying mechanisms of fibrosis in the context of transplantation and develop strategies to mitigate its negative impact, ultimately improving long-term survival and graft function for liver transplant recipients.

## Data Availability

The original contributions presented in the study are included in the article/supplementary material, further inquiries can be directed to the corresponding authors.
